# Severe RSV Infection Occurring at the End of Nirsevimab's Protection Window: A Case Report

**DOI:** 10.1155/crpe/3334926

**Published:** 2025-07-21

**Authors:** Sebastiano Mazza, Benedetta Ciccone, Anna Maddalena D'Apolito, Caterina Petruccelli, Dalila Tedeschi, Francesca Ippedico, Marianna Lauriola, Stefano Aniello Ferrante, Claudia Baiardi, Anna Calò, Francesca Fortunato, Angelo Campanozzi

**Affiliations:** ^1^Dipartimento Donna e Bambino, Azienda Ospedaliera Universitaria Foggia, Foggia, Apulia, Italy; ^2^Dipartimento di Scienze Mediche e Chirurgiche, Università Degli Studi di Foggia, Foggia, Apulia, Italy

## Abstract

We present the case of a five-month-old late preterm infant who developed severe bronchiolitis caused by respiratory syncytial virus, requiring hospitalisation and high-flow nasal cannula support. This occurred 142 days after the infant received a single dose of nirsevimab as part of the regional immunisation campaign, administered on day three of life. The patient had no underlying conditions. PCR testing revealed RSV A and human rhinovirus co-infection. The infant improved with supportive care and was discharged in stable condition after 8 days. This case raises concerns about possible waning immunity near the end of the expected 150-day protection window of nirsevimab, particularly in infants immunised early in the RSV season. Additionally, co-infection with hRV may have contributed to disease severity. Although nirsevimab remains a highly effective preventive tool, this case highlights the potential for waning immunity near the end of the protection window and suggests that ongoing surveillance is essential to optimize immunization strategies, particularly in regions with prolonged RSV seasons.

## 1. Introduction

Respiratory syncytial virus (RSV) is the most common cause of bronchiolitis in infants under 1 year of age. In this age group, it is the leading cause of death from respiratory infections and the most common cause of hospitalisation in children [1, 2]. RSV is a seasonal virus and its epidemiology varies according to climatic zones. In temperate regions of the northern hemisphere, the virus typically circulates from October to May, with a peak between December and February [1, 2]. RSV disease is recognised as a global public health priority by the World Health Organization due to its significant morbidity and mortality [1]. In 2024, there were 408 hospital admissions for RSV infections in Puglia, of which 94.4% were due to bronchiolitis, and 93% involved infants in their first year of life [3]. Between January 2011 and December 2023, a total of 1005 patients were admitted to the pediatric and neonatal units of Policlinico Foggia Hospital. Younger age (≤ 2 months) and a history of prematurity were significantly associated with increased disease severity [4].

In Italy, until recently, the standard for preventing RSV infection in preterm and high-risk infants was the monoclonal antibody palivizumab, approved in 1999, which requires up to five doses to provide protection during the peak RSV circulation period. In January 2023, nirsevimab, a new long-acting monoclonal antibody, was approved. The duration of protection provided by a single dose of nirsevimab is at least 5 months (150 days), which is considered sufficient to cover the entire RSV epidemic season and protect immunised infants from infection [1]. Two multicentre, randomised, double-blind, placebo-controlled trials—D5290C00003 (Phase IIb) and MELODY (Phase III)—evaluated the efficacy and safety of nirsevimab in preventing RSV-associated lower respiratory tract infections in term and preterm infants entering their first RSV season. In the MELODY study, after 150 days of follow-up, nirsevimab demonstrated approximately 75% efficacy in preventing medically attended lower respiratory tract infections and approximately 77% efficacy in preventing RSV-associated hospitalisations [5, 6]. Recent real-world efficacy studies have largely confirmed the data from the registrational trials. Studies from Spain, Luxembourg, France and USA report effectiveness rates between 82% and 90% in preventing RSV-associated hospitalisations in infants in their first season [7]. In the Puglia region, the passive immunisation campaign with nirsevimab was launched in October 2024. Nirsevimab was offered to all infants born during the RSV epidemic season (October 2024 to March 2025), as well as to those born between January and September 2024 who faced their first RSV season starting in October 2024. Herein, we report the case of an infant with severe bronchiolitis associated with RSV infection who required hospitalization and high-flow nasal cannula (HFNC) support, five months after immunization with nirsevimab.

## 2. Case Presentation

A 5-month-old female infant (height, 65 cm; weight, 7.15 kg) was admitted to our hospital with bronchiolitis associated with RSV infection. She was born spontaneously at 36 + 1 weeks of gestation (late preterm), with no postnatal abnormalities. Apgar score was 8 at 1 min and 9 at 5 min. Birth weight was 3050 g. Moreover, the patient had no documented history of RSV infection, no recurrent respiratory infections, and no other underlying conditions predisposing her to severe infections.

On 25 March 2025, the infant developed productive cough and clear rhinorrhea without fever. On 27 March 2025, she was seen at the Emergency Department of Foggia Hospital, where a paediatric consultation was performed. Home treatment with nebulised hypertonic saline and nasal irrigation was recommended, which provided partial relief. Two days later, she was reassessed at a pediatric outpatient clinic, where inhaled and oral corticosteroids were prescribed. Due to persistent symptoms, on 31 March 2025 another evaluation was performed, and a course of clarithromycin was initiated. Following a clinical worsening, she returned to the emergency department and was admitted to the paediatric ward on 31 March 2025 with severe respiratory distress and left apical lung consolidation shown on chest radiograph, and a diagnosis of bronchiolitis complicated by pneumonia was made. At admission, the infant presented with tachypnea (respiratory rate of 50 breaths/min) and oxygen saturation of 98% in room air. Physical examination revealed subdiaphragmatic inspiratory indentations, bilateral wheezing and widespread crackles and rales. Blood tests revealed neutrophilic leukocytosis with normal inflammatory markers (CRP: 0.80 mg/L). Within a few hours, her clinical condition worsened: respiratory rate increased to 55 breaths/min, and oxygen saturation dropped to 90% in room air. Arterial blood gas analysis showed hypoxemia (pH 7.44, pCO_2_ 34 mmHg, pO_2_ 58 mmHg). HFNC oxygen therapy was initiated with FiO_2_ of 40%, along with intravenous hydration. On the second day of hospitalisation, real-time PCR (Allplex Respiratory Panel 4) from a throat swab tested positive for RSV A and human rhinovirus (hRV). By the fifth day of hospitalization, clinical improvement was noted, including better respiratory parameters and normalized blood gas values, leading to discontinuation of HFNC therapy. The infant was discharged on the eighth day in good clinical condition.

The infant had received a single dose of nirsevimab on 9 November 2024, three days after birth, as part of the regional passive immunisation campaign. This was 142 days before diagnosis.

## 3. Discussion and Conclusions

This case describes a late preterm infant who developed severe RSV-associated bronchiolitis requiring hospitalisation and HFNC oxygen therapy approximately 5 months after receiving a single dose of nirsevimab.

The duration of the protective effect of nirsevimab is estimated to be around 150 days, which is generally the length of a typical RSV season in temperate regions. However, this case raises an important clinical consideration: infants immunised early in the epidemic season may experience waning protection in the later months, particularly if RSV circulation is prolonged.

In our case, the infant was immunised in early November 2024 and presented with severe RSV bronchiolitis at the end of March 2025, close to the upper limit of the expected duration of protection. This temporal proximity suggests that waning immunity may have contributed to the infection, despite prior prophylaxis.

This could highlight the potential limitations of a single-dose strategy for infants immunised early in the RSV season, particularly in regions where viral circulation extends into late spring, as observed in Italy and other temperate zones. Although nirsevimab remains a highly effective preventive measure [7], clinicians and public health authorities should remain aware of its time-limited protection and consider epidemiological surveillance and alternative strategies—such as flexible timing of administration or additional doses in selected cases—to extend coverage during prolonged RSV seasons.

In addition, hRV co-infection detected by PCR testing on admission may have contributed to the severity of the clinical course, as reported in paediatric populations with similar co-infection profiles [8]. Rhinovirus is the most common viral cause of respiratory infections in children and is often found in mixed viral-bacterial or viral-viral infections. It is also commonly detected in upper respiratory tract specimens, including asymptomatic children [9]. Studies report that a second virus is detected in up to 50% of RSV infections, with hRV being the most commonly identified co-pathogen. This high rate of co-infection is probably due to the overlapping seasonal circulation and high prevalence of both viruses during the winter months. The role of hRV co-infection is complex. While often implicated in more severe disease, some recent evidence suggests it may attenuate RSV severity [10]. In this case, however, its presence in an infant with severe bronchiolitis cannot be dismissed as a potential contributing factor to the overall clinical picture.

Further research is needed to assess the clinical impact of waning immunity in real-world settings and to determine whether a tailored approach to immunisation timing might optimise protection, particularly for infants immunised at the beginning of the RSV season and those with underlying conditions predisposing them to severe infections.

## Data Availability

The data that support the findings of this study are available on request from the corresponding author. The data are not publicly available due to privacy or ethical restrictions.

## Nomenclature

RSV: Respiratory syncytial virus
HFNC: High-flow nasal cannula
hRV: Human rhinovirus
FiO_2_: Fraction of inspired oxygen
PCR: Polymerase chain reaction
PaCO_2_: Partial pressure of carbon dioxide in arterial blood
PaO_2_: Partial pressure of oxygen in arterial blood
spO_2_: Oxygen saturation
CRP: C-reactive protein
